# DYRK2 Negatively Regulates Type I Interferon Induction by Promoting TBK1 Degradation via Ser527 Phosphorylation

**DOI:** 10.1371/journal.ppat.1005179

**Published:** 2015-09-25

**Authors:** Tai An, Shu Li, Wei Pan, Po Tien, Bo Zhong, Hong-Bing Shu, Shuwen Wu

**Affiliations:** 1 The College of Life Sciences, State Key Laboratory of Virology, Modern Virology Research Center, Wuhan University, Wuhan, China; 2 The College of Basic Medical Science, Shaanxi University of Chinese Medicine, Xi’an, China; The University of Chicago, UNITED STATES

## Abstract

Viral infection activates the transcription factors NF-κB and IRF3, which contribute to the induction of type I interferons (IFNs) and cellular antiviral responses. Protein kinases play a critical role in various signaling pathways by phosphorylating their substrates. Here, we identified dual-specificity tyrosine-(Y)-phosphorylation-regulated kinase 2 (DYRK2) as a negative regulator of virus-triggered type I IFN induction. DYRK2 inhibited the virus-triggered induction of type I IFNs and promoted the K48-linked ubiquitination and degradation of TANK-binding kinase 1 (TBK1) in a kinase-activity-dependent manner. We further found that DYRK2 phosphorylated Ser527 of TBK1, which is essential for the recruitment of NLRP4 and for the E3 ubiquitin ligase DTX4 to degrade TBK1. These findings suggest that DYRK2 negatively regulates virus-triggered signaling by targeting TBK1 for phosphorylation and priming it for degradation, and these data provide new insights into the molecular mechanisms that dictate the cellular antiviral response.

## Author Summary

In recent years, the mechanisms of innate antiviral immune responses mediated by pattern recognition receptors (PRRs) have been heavily investigated. All PRRs require the key molecule TANK-binding kinase 1 (TBK1) to activate the transcription factor IRF3, which leads to type I interferon induction and the cellular antiviral response. Here, we identified the dual-specificity tyrosine-(Y)-phosphorylation-regulated kinase 2 (DYRK2) as a negative regulator of TBK1. DYRK2 inhibited the virus-triggered induction of type I interferon and promoted K48-linked ubiquitination and the degradation of TBK1 in a manner that depended on its kinase activity. We further found that DYRK2 phosphorylated Ser527 of TBK1, which is essential for the recruitment of NLRP4 and for the E3 ubiquitin ligase DTX4 to degrade TBK1. Our findings suggest that DYRK2 plays an important role in innate immune responses to viruses by modulating TBK1 activity and provide important insights into the intricate regulatory mechanisms of the innate immune response against viruses.

## Introduction

The innate immune system is the first line of host defense against pathogens [1]. A limited number of germline-encoded pattern-recognition receptors (PRRs), including the Toll-like receptors (TLRs), RIG-I-like receptors (RLRs), NOD-like receptors (NLRs) and DNA receptors [1,2], recognize microbial components known as pathogen-associated molecular patterns (PAMPs) and trigger a series of signaling events that leads to the induction of type I interferons (IFNs) and proinflammatory cytokines [1,3]. Type I IFNs play central roles in antiviral responses by eliciting the expression of antiviral genes that inhibit viral replication and induce apoptotic cell death in virally infected cells, rendering the cells resistant to viral infection and activating acquired immunity [4].

All TLRs, except TLR3, recruit MyD88 and initiate MyD88-dependent signaling to activate NF-κB and MAP kinases to induce proinflammatory cytokines [2]. TLR3 and TLR4 associate with TRIF and initiate TRIF-dependent signaling [5,6]. TRIF interacts with TRAF3 and activates TBK1/IKKε, which activates IRF3/IRF7 and initiates the transcription of type I interferons [7–10]. The RLR family has three members, i.e., RIG-I, MDA5, and LGP2, which each contain a DEAD box helicase/ATPase domain, two N-terminal CARDs (LGP2 lacks the CARD) and a C-terminal regulatory domain [4]. The C-terminal regulatory domain of the RLRs is required for viral RNA recognition and binding, which induces an ATP-dependent conformational change that enables dimer or oligomer formation and exposes the CARD [11–13]. The CARDs of RIG-I and MDA5 are responsible for transmitting signals to the downstream CARD-containing adaptor VISA (also known as MAVS, IPS-1, or Cardif) through homophilic interactions between the CARDs [14–17]. VISA, which is localized at the outer mitochondrial membrane, forms large aggregates and activates the transcription factors NF-κB and IRF3/IRF7 through the IKK complex and TBK1/IKKε, respectively, resulting in the subsequent transcription of inflammatory cytokines and type I interferons [18]. Several DNA receptors have been identified, including RNA polymerase III, DAI, IFI16, DDX41, LSm14A and cGAS. Each of these DNA receptors requires a different adaptor to activate TBK1/IKKε to induce type I interferon expression in response to DNA [19–24]. In summary, PRR-induced expression of type I IFNs requires the key molecule TBK1 to activate the transcription factor IRF3.

Although type I IFNs are important for eliminating invading pathogens, the production of these cytokines needs to be properly regulated to avoid excessive harmful immune responses [25]. Ubiquitination and deubiquitination are the primary mechanisms of the regulation of TBK1 activity. The E3 ubiquitin ligase Nrdp1 and MIBs (i.e., MIB1 and MIB2) promote TBK1 activation and the transcription of type I interferon by mediating K63-linked polyubiquitination of TBK1 [26,27]. NLRP4 recruits the E3 ubiquitin ligase DTX4 to TBK1 to enable Lys48 (K48)-linked polyubiquitination, which leads to TBK1 degradation [28]. TRIP is an E3 ubiquitin ligase that also negatively regulates the cellular levels of TBK1 by directly binding to and promoting the K48-linked polyubiquitination of TBK1 [29]. Furthermore, CYLD removes K63-linked polyubiquitin from TBK1 to downregulate the IFN response [30]. Other mechanisms of the regulation of TBK1 activation include modification by phosphatases, such as SHP2, SHIP1 or PPM1B [31–33], and alterations in the function of TBK1-containing complexes, such as SIKE, TAX1BP1, zinc finger protein A20, OPTN, NEMO, TRIM11 and RNF11 [34–40]. Although it has been reported that glycogen synthase kinase 3β (GSK3β) promotes TBK1 self-association and autophosphorylation to activate IRF3 and induce IFN-b expression, this effect is not dependent on GSK3β kinase activity [41]. How TBK1 activity is negatively regulated, particularly via kinase modulation, remains largely unknown.

DYRK2 belongs to an evolutionarily conserved family of dual-specificity tyrosine-phosphorylation-regulated kinases (DYRKs) that is part of the CMGC group of protein kinases. DYRK2 contains a conserved kinase domain and an adjacent N-terminal DYRK homology (DH) box [42,43]. DYRK2 autophosphorylates a critical tyrosine residue in the activation loop and phosphorylates its substrates on serine and threonine residues [42]. Once DYRK2 is fully translated and released from the ribosome, the transitional tyrosine-kinase activity is lost, and DYRK2 subsequently functions only as a serine/threonine kinase [44]. DYRK2 appears to contribute to the regulation of xenobiotic detoxification, glucose metabolism, protein synthesis, key developmental steps, cancer progression and cellular processes via the phosphorylation of hPXR, glycogen synthase, eIF2Bε, tau, CRMP4, 4E-BP1, Snail, c-Jun, c-Myc and katanin [45–53]. Moreover, DYRK2 has also been suggested to function in various signaling pathways, including NFAT signaling in the brain and immune system, the Hedgehog signaling pathway, the hypoxia response pathway and p53 activation in response to DNA damage [54–57]. However, the exact role of DYRK2 in the virus-triggered signaling pathway has not been previously studied.

In the present study, we identified DYRK2 as a critical negative regulator of virus-triggered type I IFN signaling via the targeting of TBK1. DYRK2 phosphorylates TBK1 at S527, which is a priming event for TBK1 ubiquitination and degradation. Our findings provide a molecular mechanism for the downregulation of TBK1, which is a critical step in virus-triggered type I IFN induction and the cellular antiviral response.

## Results

### Identification of DYRK2 as a Negative Regulator of Virus-Triggered Signaling

**Fig 1:**
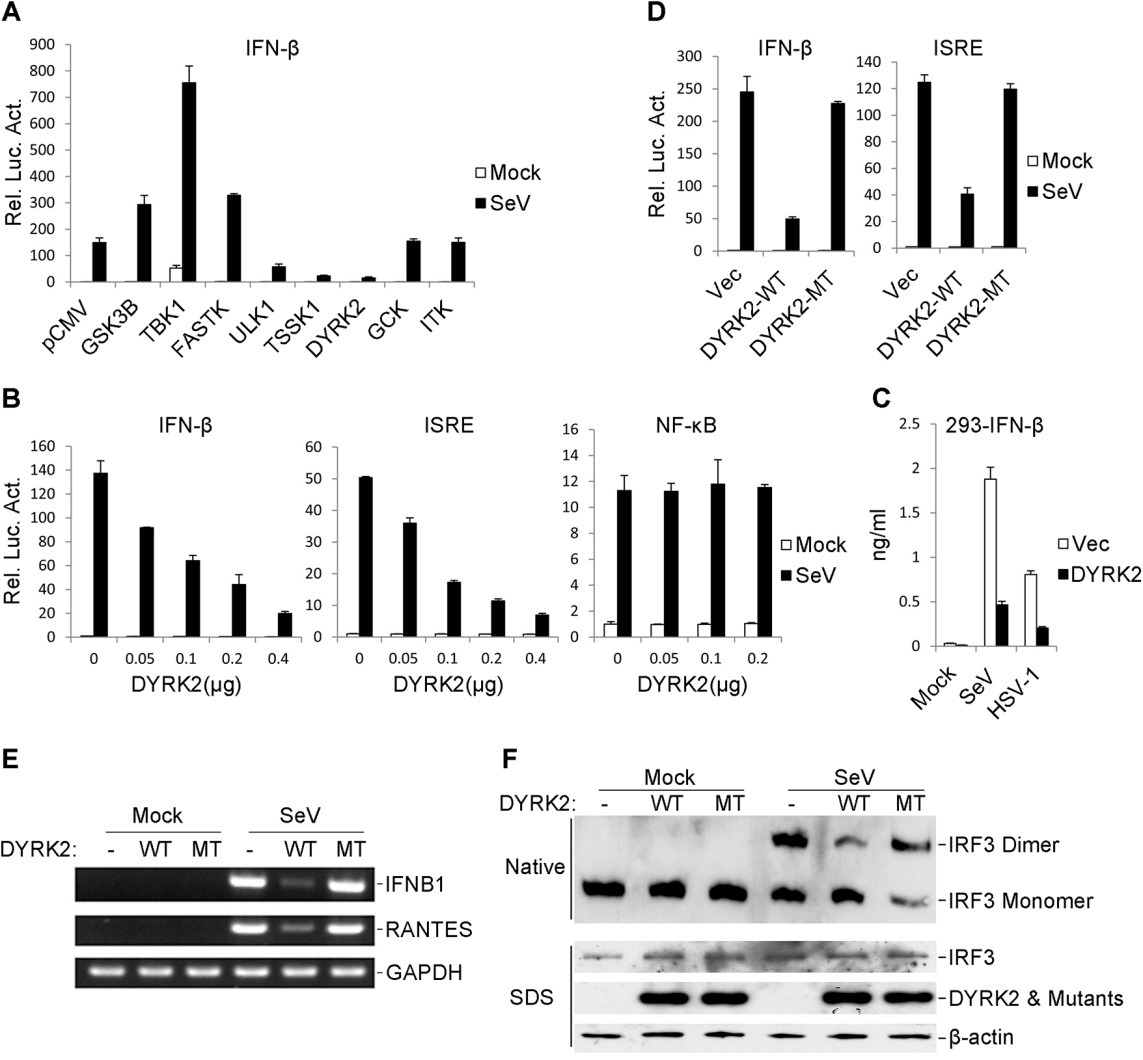
The overexpression of DYRK2 markedly inhibited the virus-triggered activations of IRF3 and IFNB1 gene transcription. (A) Identification of DYRK2 as an inhibitor of SeV-induced IFN-β activation. The 293 cells were individually transfected with human cDNA clones (Origene, Inc.) and the IFN-β luciferase reporter. Twenty hours after transfection, the cells were treated with or without SeV for 10 h before the luciferase assays were performed. (B) DYRK2 inhibited the SeV-induced activations of the ISRE and the IFN-β promoter but not NF-κB in dose-dependent manners in the 293 cells. The 293 cells (1×10^5^) were transfected with the ISRE, NF-κB reporter or IFN-β promoter luciferase plasmids (0.1 μg) and the indicated amount of DYRK2 expression plasmid. Twenty hours after transfection, the cells were infected with or without SeV for 10 h before the luciferase assays were performed. The graphical data are presented as the means ± the SDs (n = 3). (C) Effects of DYRK2 on the SeV- and HSV-1-induced secretion of IFN-β. The 293 cells (1×10^5^) were transfected with the indicated plasmids. After 20 h of incubation, the cells were infected with SeV or HSV-1 for 12 h. The culture medium was collected for quantification of the indicated cytokines by ELISA. (D) Wild-type DYRK2 but not mutant DYRK2 suppressed the SeV-induced activations of the ISRE and the IFN-β promoter. The 293 cells (1×10^5^) were transfected with the ISRE or IFN-β promoter luciferase plasmids (0.1 μg) and an expression plasmid for the wild-type DYRK2 (DYRK2-WT) or the kinase-dead DYRK2 mutant (DYRK2-MT) (0.1 μg). Twenty hours after transfection, the cells were or were not infected with SeV for 10 h before the luciferase assays were performed. The graphical data are presented as the means ± the SDs (n = 3). (E) DYRK2 inhibited the SeV-induced endogenous gene transcription of IFNB1 and RANTES. The 293 cells (2×10^5^) were transfected with the indicated plasmids (0.2 μg each) for 20 h and then infected or not infected with SeV for 10 h before reverse transcription PCR was performed. (F) DYRK2 inhibited the SeV-induced dimerization of IRF3. The 293 cells (2×10^5^) were transfected with the indicated plasmids. Twenty hours after transfection, the cells were infected with or without SeV for 10 h. Cell lysates were separated by native (top) or SDS (bottom) PAGE and analyzed by immunoblotting with the indicated antibodies.

Virus-triggered type I IFN expression is precisely regulated to prevent an excessive immune response. Because phosphorylation plays an important role in cell signaling pathways, we reasoned that virus-triggered IFN pathways may be regulated by certain kinases. To further investigate the regulation of the virus-triggered induction of type I IFNs, we screened a protein kinase cDNA library containing 347 independent expression clones using IFN-β reporter assays in 293 cells. These assays identified DYRK2 as an inhibitor of Sendai virus (SeV)-induced activation of the IFN-β promoter (Fig 1A). Further validation experiments indicated that DYRK2 inhibited the SeV-induced activation of the IFN-β promoter and the ISRE, a conserved enhancer motif that is recognized by activated IRF3, in dose-dependent manners in both 293 cells (Fig 1B) and HeLa cells (S1A and S1B Fig). However, DYRK2 overexpression did not inhibit the SeV-triggered activations of an NF-κB reporter in 293 cells (Fig 1B) or HeLa cells (S1C Fig). Similar results were observed in HeLa cells that were exposed to herpes simplex virus-1 (HSV-1) (S1D, S1E and S1F Fig). As shown in Fig 1C, the secreted IFN-β was markedly inhibited by the overexpression of DYRK2 in 293 cells. These results suggested that DYRK2 inhibited the virus-induced activation of IRF3.

A previous study determined that DYRK2 belongs to a serine/threonine protein kinase family [58]. Thus, we examined whether the kinase activity of DYRK2 was required for the virus-triggered induction of type I IFNs. As shown in Fig 1D, the kinase-dead mutant of DYRK2 (DYRK2-MT) nearly completely lost the ability to inhibit SeV-induced IFN-β activation compared to its wild-type (DYRK2-WT) counterpart. A similar conclusion was obtained upon the evaluating the SeV-triggered activation of ISRE (Fig 1D). These results suggested that kinase activity was required for DYRK2 to suppress the SeV-induced activation of ISRE and the IFN-β promoter in 293 cells. RT-PCR experiments revealed that the overexpression of DYRK2-WT but not DYRK2-MT robustly inhibited the SeV-induced gene expression of endogenous IFNB1 and RANTES in 293 cells (Fig 1E). Previous studies have shown that the induction of type I IFNs require the coordinated and cooperative actions of the transcription factors IRF3 and NF-κB [25]. The effects of DYRK2 on the virus-induced activation of IRF3 indicated that the overexpression of DYRK2-WT but not that of DYRK2-MT inhibited SeV-induced IRF3 dimerization (Fig 1F), which is a hallmark of IRF3 activation. These results suggested that DYRK2 suppressed the virus-triggered activation of IRF3 and the transcription of the IFNB1 gene and that these effects were dependent on DYRK2 kinase activity.

### Knockdown of DYRK2 Potentiates Virus-Induced IRF3 Activation and IFNB1 Transcription

**Fig 2:**
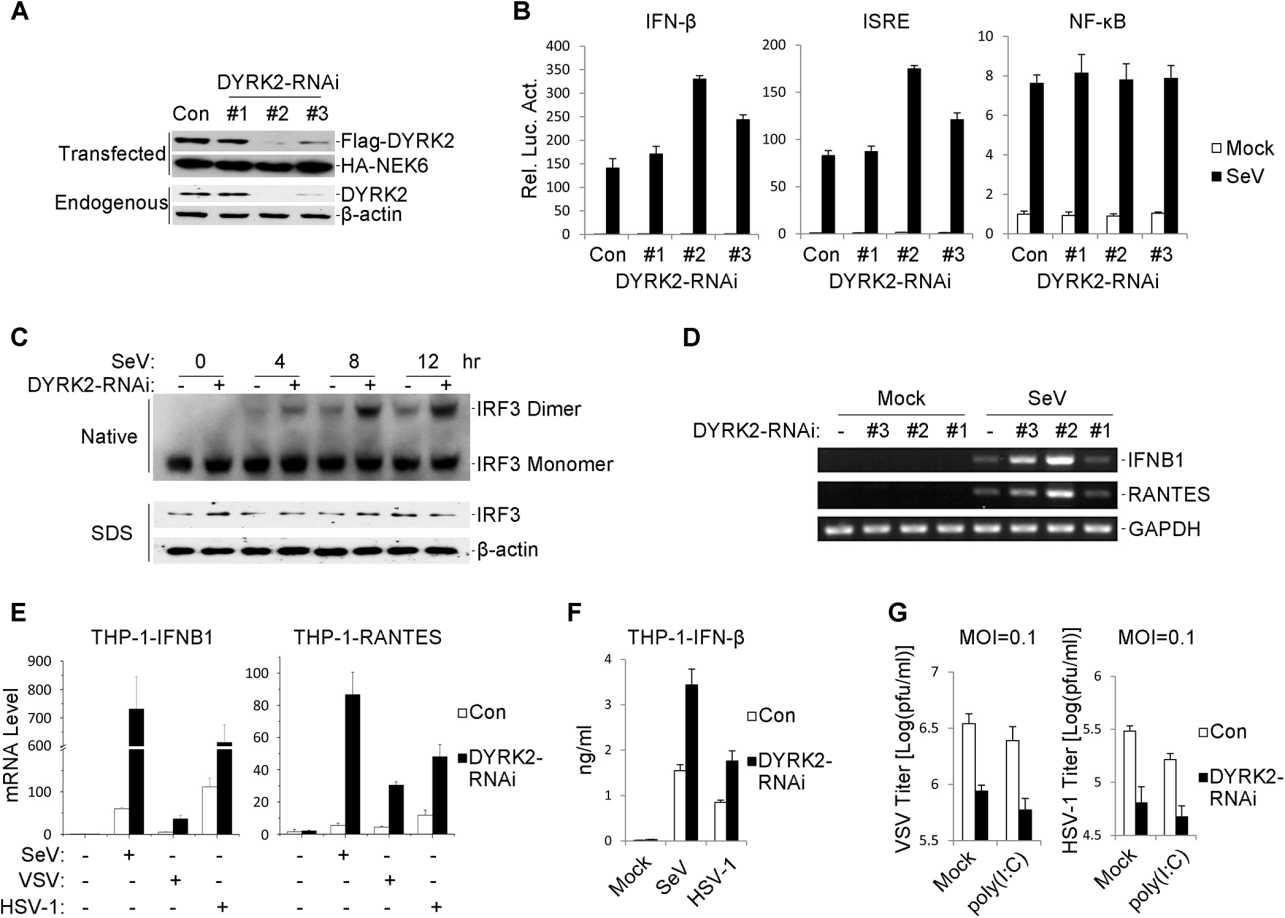
Effects of RNAi-mediated knockdown of DYRK2 on SeV-induced signaling and IRF3 activation. (A) Effects of DYRK2 RNAi on the expression of transfected and endogenous DYRK2. In the upper panel, 293 cells (2×10^5^) were transfected with expression plasmids for Flag-DYRK2 and HA-NEK6 (0.1 μg each) and the indicated RNAi plasmids (1 μg). Twenty-four hours after transfection, the cell lysates were analyzed by immunoblotting with the indicated antibodies. In the lower panel, the 293 cells (2×10^5^) were transfected with the control or indicated DYRK2 RNAi plasmids (1 μg each) for 36 h. The cell lysates were analyzed by immunoblotting with the indicated antibodies. (B) Effects of DYRK2 RNAi on the SeV-induced activations of the ISRE, NF-κB and the IFN-β promoter. The 293 cells (1×10^5^) were transfected with the ISRE, NF-κB or IFN-β promoter reporters (0.05 μg) and the indicated RNAi plasmids (0.5 μg each) for 36 h and then infected or not infected with SeV for 10 h before the luciferase assays were performed. The graphical data are presented as the means ± the SDs (n = 3). (C) Knockdown of DYRK2 promoted SeV-induced IRF3 dimerization. The 293 cells (2×10^5^) were transfected with control or DYRK2 RNAi (#2) plasmids (1 μg). Thirty-six hours after transfection, the cells were infected with or without SeV for 0, 4, 8, or 12 h. The cell lysates were separated by native (top) or SDS (bottom) PAGE, and the blots were analyzed using the indicated antibodies. (D) Effects of DYRK2 RNAi on the SeV-induced endogenous gene transcriptions of IFNB1 and RANTES. The 293 cells (2×10^5^) were transfected with the indicated RNAi plasmids (1 μg each) for 36 h and then infected or not infected with SeV for 10 h before reverse transcription PCR was performed. (E) Effects of DYRK2 RNAi on SeV-, VSV- and HSV-1-induced transcriptions of IFNB1 and RANTES in THP-1 cells. RNAi-transduced stable THP-1 cells (2×10^5^) were infected or not infected with SeV/VSV/HSV-1 for 8 h before qPCR was performed. (F) Effects of DYRK2 RNAi on SeV- and HSV-1-induced secretion of IFN-β. RNAi-transduced stable THP-1 cells (1×10^5^) were infected with SeV or HSV-1 for 12 h. The culture medium was collected for quantitation of the indicated cytokines by ELISA. (G) Effects of DYRK2 RNAi on virus replication. RNAi-transduced stable THP-1 cells (1×10^5^) were mock-transfected or transfected with poly(I:C) (1μg) for 16 h and then infected with VSV or HSV-1 (MOI = 0.1). The supernatants were harvested 24 h after infection for standard plaque assays.

We next examined whether endogenous DYRK2 was required for virus-induced signaling in physiological conditions. To accomplish this goal, we constructed three DYRK2 small hairpin (sh)RNA expressing plasmids (#1, #2 and #3) that targeted different sites within the DYRK2 mRNA. Transient transfection and endogenous expression experiments indicated that shDYRK2 #2 and #3 efficiently downregulated DYRK2 expression at the protein level as suggested by immunoblot analysis (Fig 2A). Next, we observed the effects of DYRK2 RNAi on the activation of type I interferon. Reporter assays revealed that compared with the control group, DYRK2 knockdown enhanced the activations of the IFN-β promoter and ISRE in 293 cells (Fig 2B) and HeLa cells (S2A, S2B, S2D and S2E Fig) in response to SeV and HSV-1. Consistently, DYRK2 knockdown did not affect the virus-induced activations of NF-κB in 293 cells (Fig 2B) or HeLa cells (S2C and S2F Fig).

To further assess the effects of DYRK2 RNAi on the endogenous gene expressions of IFNB1 and RANTES, we knocked down DYRK2 in 293 cells and then infected the cells with or without SeV. SeV infection resulted in increased mRNA expressions of IFNB1 and RANTES in the cells that were transfected with shDYRK2-expressing plasmid as compared with the control transfected cells (Fig 2D). Because the effect of shDYRK2 #2 elicited the greatest results of the three RNAi sequences (see Fig 2A, 2B and 2D), the #2 plasmid was used for all of the following experiments. The induction of IFNB1 gene transcription requires the activation of the transcription factor IRF3. We next determined whether DYRK2 RNAi affected the virus-induced activation of IRF3. DYRK2 knockdown clearly potentiated SeV-induced IRF3 dimerization (Fig 2C). qPCR experiments further confirmed that IFNB1 and RANTES mRNA expression were induced by SeV; VSV and HSV-1 were markedly higher in the DYRK2 RNAi-transduced stable THP-1 cells (Fig 2E). Compared with the control groups, the secreted IFN-β proteins were markedly higher in the DYRK2 knockdown THP-1 cells following infection with SeV and HSV-1 (Fig 2F). Because DYRK2 negatively regulated the virus-triggered IFN-β expression, we next determined whether DYRK2 was involved in the regulation of the cellular antiviral response. Plaque assays indicated that knockdown of DYRK2 significantly inhibited VSV and HSV-1 replication and further promoted the cytoplasmic poly (I:C)-mediated inhibition of VSV and HSV-1 replication (Fig 2G). Together, these results implied that DYRK2 was a physiological inhibitor of virus-triggered IFN-β induction and the cellular antiviral response.

### DYRK2 Regulated Virus-Induced Signaling by Targeting TBK1

**Fig 3:**
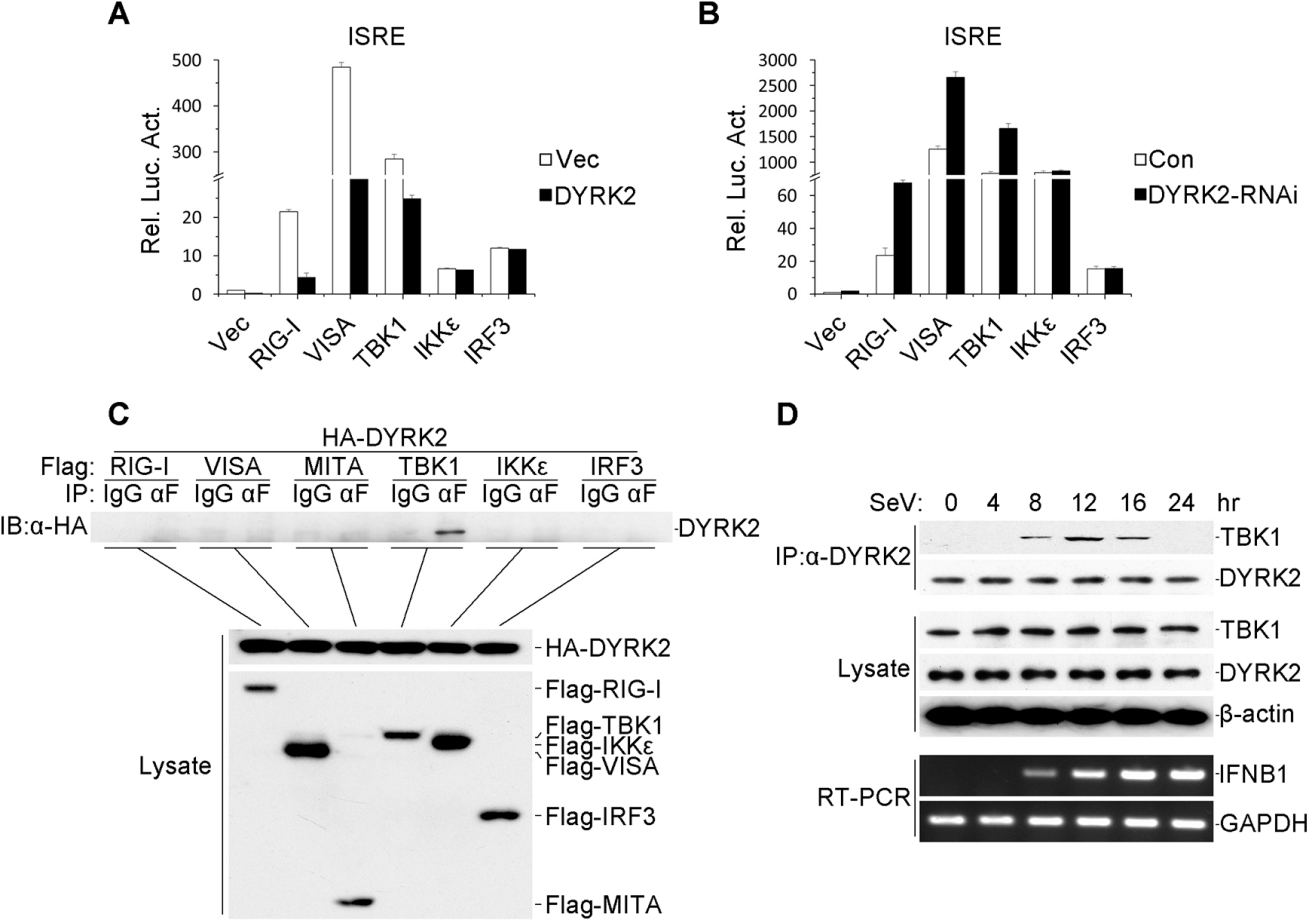
DYRK2-mediated virus-triggered signaling at the level of TBK1. (A) The overexpression of DYRK2 inhibited RIGI-, VISA-, and TBK1-mediated signaling but not IKKε- and IRF3-mediated signaling. The 293 cells (1×10^5^) were transfected with DYRK2 or control plasmids (0.1 μg) together with ISRE luciferase and the indicated plasmids (0.1 μg each). Luciferase assays were performed 20 h after transfection. The graphical data are presented as the means ± the SDs (n = 3). (B) Knockdown of DYRK2 promoted ISRE activation by RIGI, VISA, and TBK1 but not by IKKε or IRF3. The 293 cells (1×10^5^) were transfected with control or DYRK2 RNAi (#2) plasmids (0.5 μg). After 20 h, the cells were selected with puromycin (1 μg/ml) for 24 h and then re-transfected with the ISRE luciferase and the indicated expression plasmids (0.1 μg each). Reporter assays were performed 24 h after transfection. The graphical data are presented as the means ± the SDs (n = 3.) (C) DYRK2 interacted with TBK1 but not with RIG-I, VISA, MITA, IKKε, or IRF3 in the overexpression system. The 293 cells (2×10^6^) were transfected with the indicated plasmids (5 μg each). Coimmunoprecipitation and immunoblotting analyses were performed with the indicated antibodies (upper). The expressions of the transfected proteins were analyzed by immunoblotting with anti-HA or anti-Flag antibodies (lower). (D) Kinetics of the DYRK2–TBK1 association after viral infection. The 293 cells (1×10^8^) were treated with MG132 and infected with SeV for the indicated times. Small fractions of the cells were prepared for RT-PCR (lower). The remaining cell fractions were lysed, and the lysates were coimmunoprecipitated with an anti-DYRK2 antibody or preimmune serum. The coimmunoprecipitates were analyzed by immunoblotting with anti-TBK1 or anti-DYRK2 antibodies (upper). The expression levels of endogenous TBK1, DYRK2, and β-actin were detected by immunoblot analyses with the indicated antibodies (middle).

As described above, the overexpression and endogenous experiments revealed that DYRK2 was required for and acted as an inhibitor in virus-triggered signaling. Next, we sought to determine the levels at which DYRK2 regulated the virus-induced IRF3 activation pathway. To accomplish this goal, the 293 cells were cotransfected with plasmids encoding DYRK2 and signaling components. DYRK2 dramatically inhibited the RIG-I-, VISA- and TBK1-mediated but not the IKKε- or IRF3-mediated activation of the ISRE (Fig 3A). Conversely, DYRK2 knockdown elicited the opposite results (Fig 3B). These results suggested that DYRK2 may negatively regulate virus-triggered signaling by interacting with TBK1.

To further confirm the interactions between DYRK2 and TBK1, transient transfection and coimmunoprecipitation experiments were performed, and the data revealed that DYRK2 associated with TBK1 but not with other signaling molecules, including RIG-I, VISA, MITA, IKKε and IRF3 (Fig 3C). Although TBK1 and IKKε have redundant roles in certain circumstances, it appeared that DYRK2 only targeted TBK1 and not IKKε (Fig 3A, 3B and 3C), which indicated that DYRK2 specifically interacted with TBK1.

We also determined whether DYRK2 physically interacted with TBK1 in untransfected cells. We performed endogenous coimmunoprecipitation and immunoblotting analyses with SeV- and MG132-treated cells at various time points. As shown in Fig 3D, DYRK2 did not associate with TBK1 under physiological conditions. However, the interaction between DYRK2 and TBK1 became evident 8 hours after infection, gradually increased between 8 and 12 hours after stimulation, and then completely disappeared. Interestingly, RT-PCR experiments revealed that the IFN-β mRNA expression induced by SeV infection gradually increased, reaching a maximum 16 hours after infection (Fig 3D). These results implied that DYRK2 associated with TBK1 in a viral infection-dependent manner and that this interaction limited virus-induced IFN-β transcription.

### DYRK2 Promoted TBK1 Degradation via K48-Linked Ubiquitination

**Fig 4:**
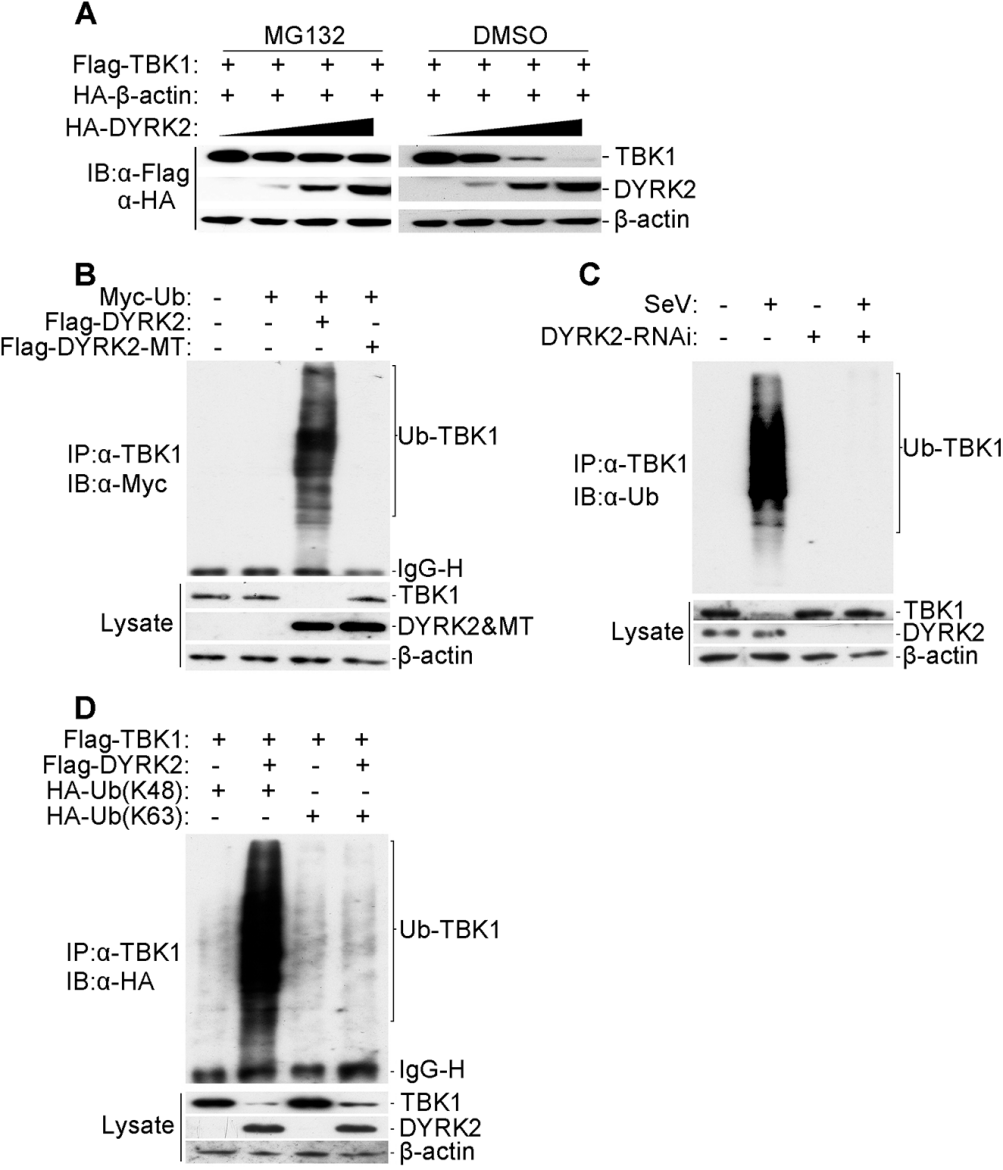
DYRK2 promoted TBK1 degradation via K48-linked ubiquitination. (A) Overexpression of DYRK2 induced TBK1 degradation in a dose-dependent manner. The 293T cells (2×10^5^) were transfected with the Flag-TBK1, HA-β-actin and HA-DYRK2 plasmids (0.1, 0.2 or 0.4 μg) and were treated with dimethyl sulfoxide (DMSO) or MG132. The cells were lysed, and the lysates were analyzed by immunoblotting with anti-Flag or anti-HA antibodies. (B) Overexpression of wild-type DYRK2 but not mutant DYRK2, promoted the ubiquitination of TBK1. The 293 cells (1×10^7^) were transfected with the indicated plasmids. Twenty-four hours after transfection, cell lysates were immunoprecipitated with an anti-TBK1 antibody. The immunoprecipitates were analyzed by immunoblotting with an anti-Myc antibody (upper). Protein expression was analyzed by immunoblotting with the indicated antibodies (lower). (C) Effects of DYRK2 RNAi on the SeV-induced ubiquitination of endogenous TBK1. The 293 cells (5×10^7^) were transfected with control or DYRK2 RNAi (#2) plasmids. Twenty hours after transfection, the cells were infected or not infected with SeV for 10 h. The cell lysates were immunoprecipitated with an anti-TBK1 antibody. The immunoprecipitates were analyzed by immunoblotting with an anti-ubiquitin antibody (top). The expressions of related proteins were examined by immunoblotting with the indicated antibodies (bottom). (D) DYRK2 promoted K48-linked but not K63-linked ubiquitination of TBK1. The 293 cells (2×10^6^) were transfected with HA-tagged Lys-48-only or Lys-63-only ubiquitin plasmids and the other indicated plasmids. Twenty-four hours after transfection, cell lysates were immunoprecipitated with an anti-TBK1 antibody and then analyzed by immunoblotting with an anti-HA antibody (upper panel). The expressions of related proteins were examined by immunoblotting with the indicated antibodies (lower panel).

We next determined how DYRK2 regulated the virus-trigged induction of IFN-β via its interaction with TBK1. To achieve this goal, we cotransfected the plasmid encoding Flag-tagged TBK1 with increasing amounts of the plasmid encoding HA-tagged DYRK2 into 293 cells, and the cells were then treated with dimethyl sulfoxide (DMSO) or the proteasome inhibitor MG132. With increased DYRK2 expression, TBK1 levels gradually decreased (Fig 4A). In contrast, no change in TBK1 expression was associated with the increased DYRK2 expression in MG132-treated cells (Fig 4A). These results suggested that DYRK2 promoted TBK1 degradation in a dose-dependent manner and that this process was proteasome-dependent.

We then determined whether DYRK2 elicited the ubiquitination of TBK1. Fig 4B shows that DYRK2-WT but not DYRK2-MT caused the ubiquitination and downregulation of TBK1. As the interactions between DYRK2 and TBK1 were viral infection-dependent under physiological conditions (Fig 3D), we also wanted to determine whether viral infection caused the ubiquitination of endogenous TBK1. Endogenous TBK1 was ubiquitinated and downregulated after virus infection (Fig 4C). Conversely, DYRK2 knockdown markedly diminished the virus-induced ubiquitination and downregulation of TBK1 (Fig 4C). These results illustrated that DYRK2 targeted TBK1 for ubiquitination and degradation after virus infection.

Because proteasomes normally recognize and degrade proteins that have been modified with K48-linked polyubiquitin chains [59], we also determined whether DYRK2 promoted the K48- or K63-linked ubiquitination of TBK1. To achieve this goal, plasmids expressing ubiquitin mutants containing a single lysine residue, i.e., K48 (ubiquitin-K48) or K63 (ubiquitin-K63), were used. Immunoprecipitation and immunoblot analyses revealed that TBK1 was predominantly modified by K48-linked ubiquitination and less so by K63-linked ubiquitination in cells cotransfected with DYRK2 and TBK1 compared with cells transfected with TBK1 alone (Fig 4D). Collectively, these results demonstrated that DYRK2 specifically induced the K48-linked ubiquitination of TBK1, which is recognized by and subsequently degraded by the proteasome pathway.

### DYRK2 Catalyzed TBK1 Phosphorylation at Ser527

**Fig 5:**
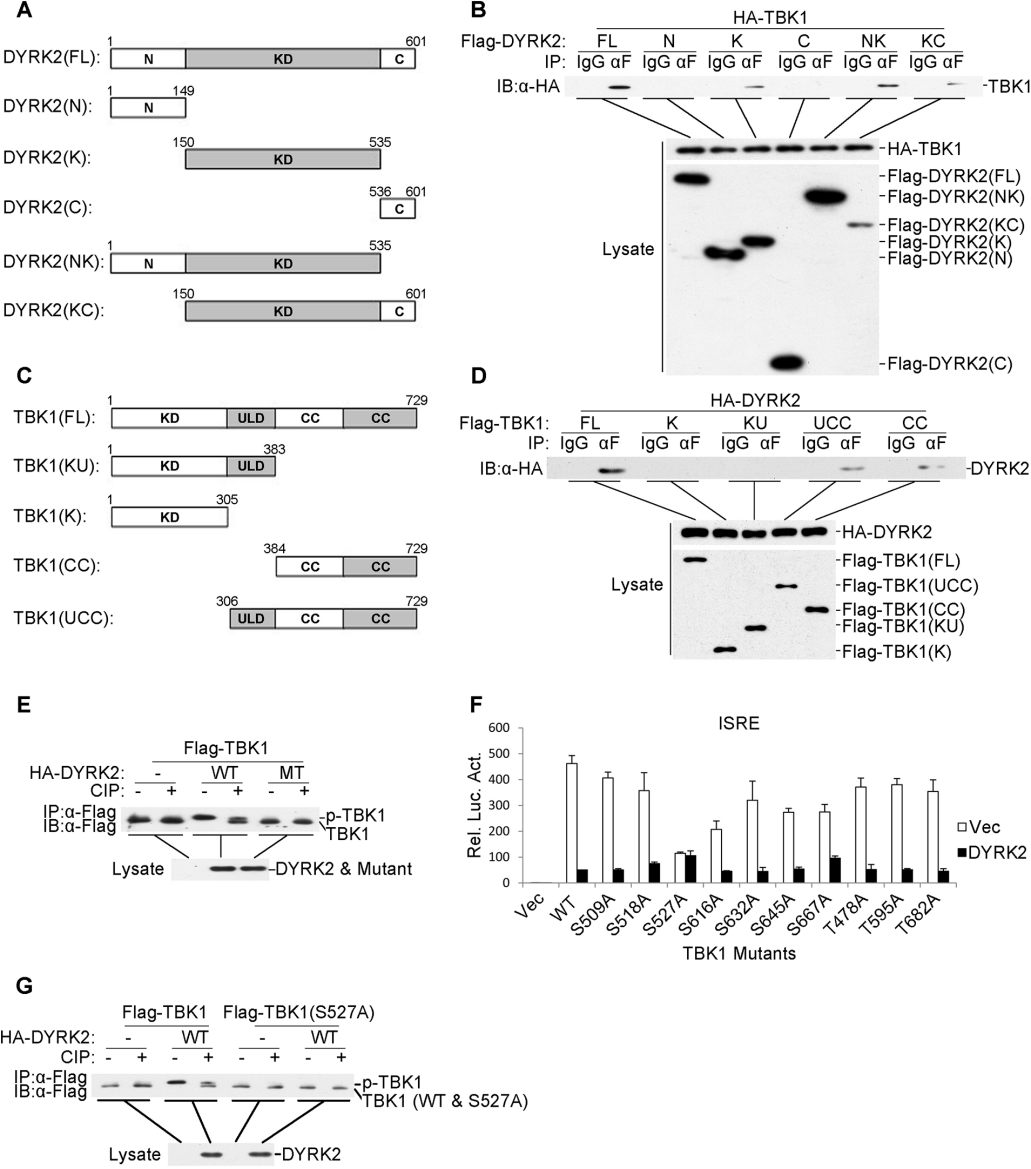
DYRK2 phosphorylated TBK1 at S527. (A and C) Schematics of human DYRK2 (A) and TBK1 (C) and their truncation mutants. (B) DYRK2 interacted with TBK1 via its kinase domain. The 293 cells (2×10^6^) were transfected with the indicated plasmids (5 μg each). Coimmunoprecipitations were performed with anti-Flag or IgG (control). The immunoprecipitates were analyzed by immunoblotting with an anti-HA antibody (top). The expressions of the transfected proteins were analyzed by immunoblotting with anti-HA (middle) or anti-Flag (bottom) antibodies. (D) DYRK2 bound to the coiled-coil domain of TBK1. The 293 cells (2×10^6^) were transfected with the indicated plasmids and then treated and analyzed as described in (B). (E) Wild-type DYRK2 but not mutant DYRK2 promoted TBK1 phosphorylation. The 293 cells (2×10^6^) were transfected with the indicated plasmids (5 μg each). Cell lysates were immunoprecipitated with an anti-Flag antibody, treated with or without calf intestine phosphatase (CIP), and analyzed by immunoblotting with an anti-Flag antibody (top). The expressions of the transfected proteins were analyzed by immunoblotting with an anti-HA antibody (bottom). (F) The effects of DYRK2 on the wild-type and mutant TBK1 as determined by ISRE activation. The 293 cells (1×10^5^) were transfected with an ISRE reporter plasmid (0.1 μg) and the indicated expression plasmids (0.1 μg each) for 20 h before the luciferase assays were performed. The graphical data are presented as the means ± the SDs (n = 3). (G) DYRK2 promoted the phosphorylation of wild-type TBK1 but not the TBK1 S527A mutant. The 293 cells (2×10^6^) were transfected with the indicated plasmids and then treated and analyzed as described in (E).

DYRK2 contains a canonical kinase domain that is located between a large N-terminal domain (149 amino acids) and a short C-terminal extension (66 amino acids) [43]. To determine which domain of DYRK2 was necessary for TBK1 ubiquitination and degradation, we constructed five DYRK2 deletion mutants (Fig 5A). Coimmunoprecipitation experiments indicated that all of the deletion mutants of DYRK2 associated with TBK1, except those that only contained the N- or C-terminal domains (Fig 5B), suggesting that the DYRK2 kinase domain directly interacted with TBK1. We also generated TBK1 deletion mutants containing various combinations of the TBK1 domains for coimmunoprecipitation analyses (Fig 5C). Among these mutants, only the truncated proteins containing the coiled-coil domain interacted with DYRK2, whereas the mutants that only containing the kinase or/and ubiquitin domains did not interact with DYRK2 (Fig 5D). These results demonstrated that DYRK2 utilized its kinase domain to bind to the coiled-coil domain of TBK1.

As described above, DYRK2 inhibited the virus-induced activation of IRF3 and the transcription of IFN-β and that these effects depended on its kinase activity (Fig 1). Additionally, DYRK2 interacted with TBK1 through its kinase domain (Fig 5B). Therefore, we hypothesized that DYRK2 regulated the activity of TBK1 via phosphorylation. To confirm this theory, we performed immunoprecipitation assays and determined that the cotransfection of wild-type (WT) DYRK2 but not the kinase-dead mutant (MT) with TBK1 caused TBK1 to migrate to a position that was associated with an increased molecular weight (Fig 5E). This higher band represented phosphorylated TBK1 because it could be digested into two bands by calf intestine phosphatase (CIP) (Fig 5E). One band represented phosphorylated TBK1, which was incompletely digested by CIP (higher band), and the other band represented CIP-dephosphorylated TBK1 (lower band) (Fig 5E).

We next sought to determine which sites within TBK1 were phosphorylated and therefore affected TBK1 function. We constructed a series of point mutants of TBK1 at the potential phosphorylation residues that were predicted with the NetPhos program; these point mutant constructs were cotransfected with DYRK2 into 293 cells, and the activations of ISRE were analyzed. As shown in Fig 5F, DYRK2 clearly inhibited the activation of the ISRE that was mediated by all the mutants except the S527A mutant. We then examined the DYRK2 phosphorylation of TBK1S527A; DYRK2 only phosphorylated TBK1 wild-type (WT) and not TBK1 (S527A) (Fig 5G). To acquire more evidence, phosphomimetic TBK1 S527D and S527E mutants were constructed. As shown in S3A Fig, the TBK1 mutants of S527D and S527E were constitutively ubiquitinated and degraded regardless of the presence of DYRK2. Reporter assays revealed thatDYRK2 had no effect on the activations of the IFN-β promoter that were mediated by any of the TBK1 mutants (S3B Fig). qPCR results revealed that the expressions of the endogenous IFNB1, RNATES and ISG15 mRNAs were not inhibited by DYRK2 when the cells were stimulated with the TBK1 mutants (S3C Fig). It should be noted that TBK1(S527A) did not activate the IFN-β promoter or the expression of downstream genes as robustly as did wild-type TBK1. The reason behind the phenomenon is unclear. It is possible that mutation of Ser527 into Ala disrupt the structure of TBK1 which impairs its function to phosphorylate IRF3. In the plaque assays, we found that the overexpression of DYRK2 markedly promoted VSV replication in the cells that were transfected with wild type TBK1 and that this promotion effect disappeared in the cells that were transfected with mutant TBK1 (S3D Fig). These results indicated that DYRK2 specifically phosphorylated TBK1 at the Ser527 residue.

### DYRK2 was Essential for the NLRP4-Mediated Inhibition of Type I Interferon Induction

**Fig 6:**
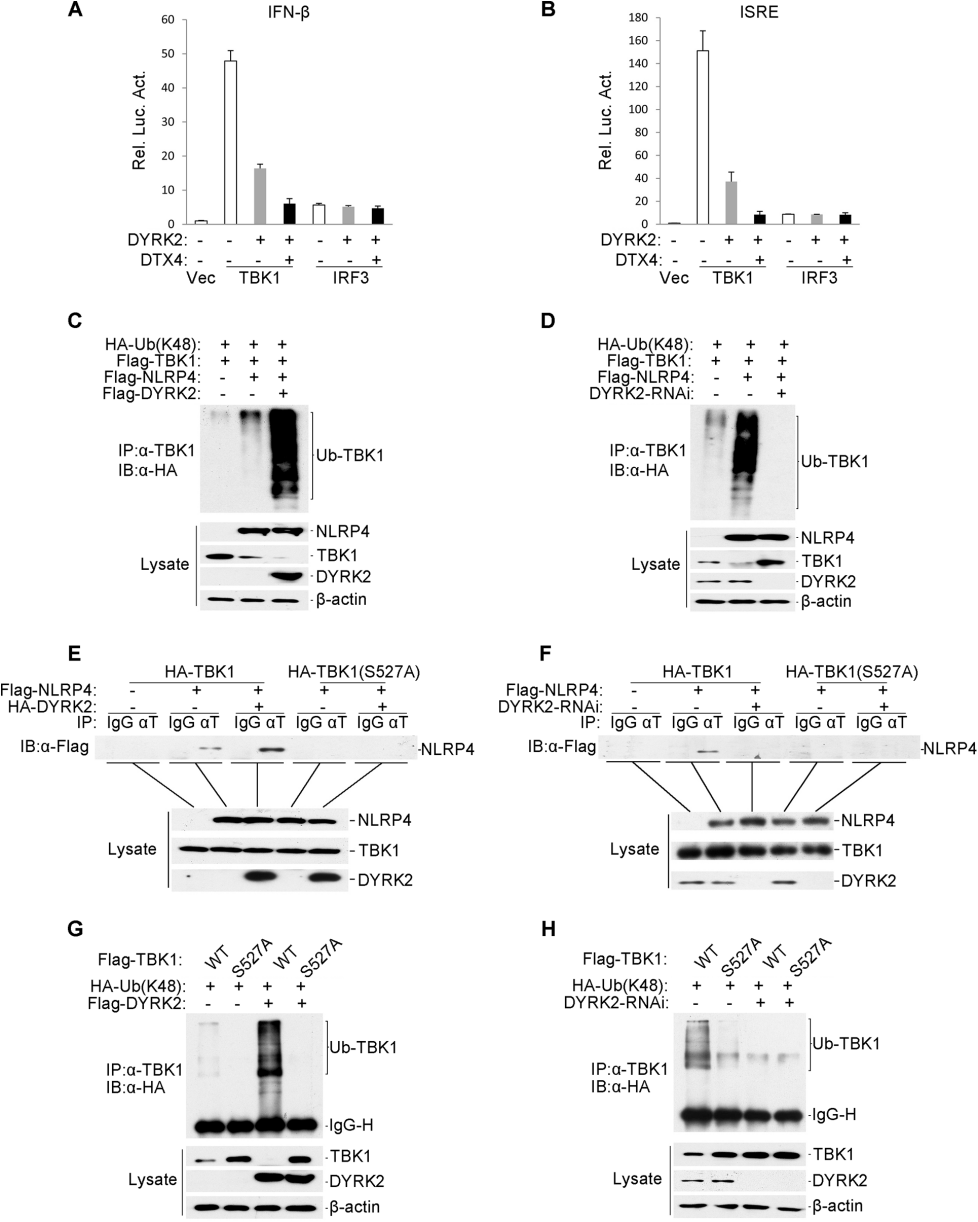
Effects of DYRK2 on the NLRP4-mediated inhibition of Type I interferon induction. (A and B) The synergistic inhibition of DTX4 on the activation of the IFN-β promoter (A) and ISRE (B). The 293 cells (1×10^5^) were transfected with the indicated plasmids. Luciferase assays were performed 20 h after transfection. The graphical data are presented as the means ± the SDs (n = 3). (C and D) DYRK2 overexpression potentiated (C) whereas DYRK2 knockdown inhibited (D) the degradation of TBK1 that was induced by NLRP4. The 293 cells (2×10^6^) were transfected with the indicated plasmids. Twenty-four hours after transfection, cell lysates were immunoprecipitated with an anti-TBK1 antibody. The immunoprecipitates were analyzed by immunoblotting with an anti-HA antibody (upper). Protein expression was analyzed by immunoblotting with the indicated antibodies (lower). (E and F) DYRK2 overexpression enhanced (E) whereas DYRK2 knockdown abolished (F) the interaction between TBK1 and NLRP4. The 293 cells (2×10^6^) were transfected with the indicated plasmids and then treated with (E) or without (F) MG132. Cell lysates were analyzed with the indicated antibodies (upper). The expression of the transfected proteins was analyzed by immunoblotting with anti-HA and anti-Flag antibodies (lower). (G and H) DYRK2 promoted the degradation of wild-type TBK1 but not of the S527A mutant. The 293 cells (2×10^6^) were transfected with the indicated plasmids and then treated and analyzed as described in (C and D).

We showed that DYRK2 not only phosphorylated serine 527 of TBK1 (Fig 5G) but also promoted the K48-linked ubiquitination of TBK1 and its subsequent degradation (Fig 4D). Next, we sought to determine how DYRK2 promoted the ubiquitination of TBK1 through phosphorylation. We screened a cDNA array containing 352 expression clones of ubiquitin-related enzymes by cotransfecting TBK1 with each clone into cells overexpressing DYRK2 and found that the E3 ubiquitin ligases DTX4 and DYRK2 synergistically inhibited the TBK1-mediated activation of the ISRE and the IFN-β promoter (Fig 6A and 6B). It has been reported that NLRP4 recruits the E3 ligase DTX4 to TBK1 for K48-linked ubiquitination, which leads to TBK1 degradation [28]. To further confirm the function of DYRK2 in the NLRP4-mediated degradation of TBK1, we performed an immunoprecipitation analysis. DYRK2 overexpression significantly enhanced the NLRP4-induced K48-linked ubiquitination of TBK1 and its subsequent degradation (Fig 6C). Consistently, in the context of DYRK2 knockdown, NLRP4 did not induce the K48-linked ubiquitination or degradation of TBK1 (Fig 6D). These results indicated that DYRK2 plays a critical role in NLRP4-mediated TBK1 polyubiquitination and degradation. Coimmunoprecipitation experiments revealed that DYRK2 overexpression enhanced the interaction between NLRP4 and TBK1 (Fig 6E) and that DYRK2 knockdown abolished this interaction (Fig 6F). Furthermore, the TBK1 S527A mutant did not interact with NLRP4 (Fig 6E and 6F). We previously determined that the Ser527 of TBK1 is phosphorylated by DYRK2 (Fig 5G). Here, the TBK1 S527A mutant could not be ubiquitinated when DYRK2 was overexpressed (Fig 6G), and wild-type TBK1 could not be ubiquitinated when DYRK2 was knocked down (Fig 6H). These results suggested that DYRK2 was necessary for the interaction between NLRP4 and TBK1 and that the phosphorylation of TBK1 at Ser527 by DYRK2 determined whether NLRP4 could interact with TBK1 and elicit subsequent TBK1 ubiquitination and degradation.

## Discussion

The virus-triggered induction of type I interferon is regulated in a spatio-temporal manner by various molecules and distinct mechanisms [18,60]. Although it is clear that TBK1 plays an essential role in antiviral responses, the mechanisms by which its activities are regulated are unknown. In this study, we identified DYRK2 as a novel negative regulator of the virus-induced type I interferon induction pathway and determined that the mechanism of action of DYRK2 involved the phosphorylation of Ser527 on activated TBK1 followed by the triggering of K48-linked ubiquitination and degradation mediated by the NLRP4-DTX4 complex.

DYRK2 is involved in regulating numerous cellular signal transduction pathways, such as NFAT signaling, Hedgehog signaling and the hypoxia response and p53-activated signaling pathways [54–57]. Our results indicated that DYRK2 was essential for virus-triggered IFN-β induction and the cellular antiviral response and that this process was dependent on its kinase activity. Ectopic expression of DYRK2 inhibited the activations of the ISRE and the IFN-β reporter, the dimerization of IRF3, and the transcription and secretion of INF-β, whereas the kinase-dead mutant of DYRK2 (DYRK2-MT) abolished this inhibitory activity. Conversely, RNAi-mediated knockdown of DYRK2 potentiated the virus-triggered activations of IRF3, IFNB1 and RANTES gene expressions and the secretion of INF-β and inhibited viral replication.

A series of experiments indicated that DYRK2 targeted TBK1 and facilitated TBK1 degradation under physiological conditions. First, the reporter assays revealed that DYRK2 markedly inhibited the RIG-I-, VISA-, and TBK1-mediated but not the IKKε- and IRF3-mediated activations of the ISRE, whereas DYRK2 knockdown had the opposite results. These findings are consistent with the molecular placement of TBK1 within the pathway. Second, DYRK2 specifically bound to TBK1 in mammalian overexpression systems. Third, the interaction between DYRK2 and TBK1 was the strongest at twelve hours after viral infection under physiological conditions; the interaction subsequently gradually disappeared. The kinetics of the disassociation between these two proteins correlated with the termination of IFNB1 gene transcription. Fourth, compared with the MG132-treated control group, DYRK2 promoted TBK1 degradation in a dose-dependent manner. Finally, overexpression experiments revealed that DYRK2 effectively promoted the K48-linked ubiquitination of TBK1 and its subsequent degradation.

DYRK2 belongs to the Ser/Thr protein kinase family [61], implying that DYRK2 might phosphorylate TBK1. Our results indicated that the kinase domain (KD) of DYRK2 interacted with the coiled-coil domain (CC) of TBK1. We further demonstrated that DYRK2 specifically phosphorylated Ser527 of TBK1 by point mutation analysis. DYRK2 requires an arginine at the -2 or -3 position to efficiently phosphorylate its substrates, and replacing the +1 proline with an alanine nearly completely eliminates substrate phosphorylation [62]. Interestingly, the sequence around Ser527 of TBK1 (RL**S**P) coincides with the consensus DYRK2 phosphorylation site. Crosstalk between different types of posttranslational modifications, particularly between phosphorylation and ubiquitination, is an emerging theme in eukaryotic cells [63]. Although previous studies have suggested that NLRP4 recruits the E3 ubiquitin ligase DTX4 to TBK1 for Lys48 (K48)-linked polyubiquitination and the degradation of TBK1 [28], the initiating event is unclear; in other words, there must be a signal that initiates this sequence. In our study, we proved that DYRK2 overexpression clearly promoted the NLRP4-mediated K48-linked polyubiquitination and degradation of TBK1, whereas DYRK2 knockdown failed to induce the polyubiquitination and degradation of TBK1. Furthermore, DYRK2 knockdown eliminated the interaction between NLRP4 and TBK1, whereas DYRK2 overexpression enhanced this interaction. Furthermore, the TBK1 S527A mutant could not be ubiquitinated in the context of overexpressed DYRK2, whereas wild-type TBK1 was ubiquitinated. Additionally, wild-type TBK1 could not be ubiquitinated when DYRK2 was knocked down. Together, these data suggested that DYRK2-mediated phosphorylation was a priming event that was required for the NLRP4-mediated K48-linked polyubiquitination and degradation of TBK1.

Based on all of our findings, we propose the following hypothesis to explain how DYRK2 negatively regulates the type I interferon induction pathway. In uninfected cells, TBK1 does not interact with DYRK2. Once a cell is infected, TBK1 is rapidly activated by upstream signaling, which activates IRF3 and type I interferon expression to fight the invading virus. To prevent excessive harmful immune responses, DYRK2 may be activated by a similar mechanism mediated by ATM [64] and bind to TBK1 via its kinase domain and phosphorylate Ser527 at the appropriate time after virus infection. Only phosphorylated TBK1 is recognized by and binds to NLRP4. Ultimately, NLRP4 recruits the E3 ubiquitin ligase DTX4 to degrade TBK1 via K48-linked ubiquitination, and signal transduction is terminated. In this regulation process, it remains unclear how phosphorylated TBK1 is specifically recognized by NLRP4. A previous report indicated that DYRK2 is required for the assembly of the DYRK2-EDVP E3 ligase complex and phosphorylates its substrates to prime them for degradation [53]. The functions of DYRK2, NLRP4 and DTX4 and whether a DYRK2-EDVP complex functions in the regulation of the activity of TBK1 require further study. In conclusion, our findings revealed that DYRK2 phosphorylates TBK1 at Ser527 and subsequently triggers the ubiquitination and degradation of TBK1, which provides new insight into the mechanism of the control of excessive cellular antiviral responses.

## Materials and Methods

### Reagents

This study utilized antibodies against TBK1 (Cell Signaling Technology), IRF3, DYRK2, ubiquitin (Santa Cruz Biotechnology), Flag, HA, Myc, β-actin (Sigma-Aldrich), and horseradish peroxidase (HRP)-conjugated anti-rabbit IgG and anti-mouse IgG (Thermo Fisher Scientific). Mouse antisera against TBK1 and DYRK2 were raised against the respective recombinant human proteins. The human IFN Beta ELISA kits (Pestka Biomedical Laboratories) and SYBR qPCR Mix (TOYOBO) were purchased from the indicated companies. SeV, VSV, and HSV-1 were prepared as previously described [23].

### Constructs

The ISRE and IFN-b promoter luciferase reporter plasmids and mammalian expression plasmids for HA- or Flag-tagged RIG-I, VISA, MITA, TBK1, IKKε, and IRF3 have been previously described [65]. The mammalian expression plasmids for HA-tagged Lys-48- and Lys-63-only ubiquitin mutants were made by site-directed mutagenesis [59]. The mammalian expression plasmids for human DYRK2, NLRP4 and DTX4 were purchased from OriGene. The mammalian expression plasmids for wild-type and mutant Flag- and HA-tagged DYRK2 were constructed with standard molecular biology techniques. TBK1 mutants were provided by Cao-Qi Lei (Wuhan University).

### Transfection and Reporter Gene Assays

The transfection and reporter assays were performed as previously described [66]. The 293 cells were cultured in 24-well plates and transfected with the indicated plasmids on the following day by standard calcium phosphate precipitation. In these experiments, the pRL-TK (Renilla luciferase) reporter plasmid was added into each well to normalize the transfection efficiency, and the empty vector plasmid was used to ensure that the same amounts of total DNA were transfected into each well. Twenty hours after transfection, the cells were stimulated with or without SeV for 10 hours in certain experiments; otherwise, the cells were harvested, and the luciferase assays were performed using a dual-specific luciferase assay kit (Promega). Firefly luciferase activity was normalized to Renilla luciferase activity. All of the reporter assays were repeated three times.

### PCR

Total RNA was extracted from cells using TRIzol reagent (Invitrogen) following the protocols recommended by the manufacturer. The RNA samples were then treated with Ambion Turbo RNA-free DNase I (Ambion) at 37°C for 30 min to remove the residual DNA. cDNA was prepared from 2 μg of RNA using oligo(dT), M-MLV reverse transcriptase (Promega), and RNasin Ribonuclease Inhibitor (Biostar International) in a total volume of 25 μl at 37°C for 1 h. cDNA was subjected to semi-quantitative PCR analysis to measure the expressions of IFNB1, RANTES and GAPDH according to the manufacturer’s instructions. For each qPCR reaction, the samples were mixed with the Thunderbird SYBR qPCR Mix (TOYOBO), and the final primer concentrations were 0.3 μM. The amplifications were performed in a qPCR system (ABI 7300). The conditions included 1 cycle of 95°C for 1 min, followed by 40 cycles of 95°C for 15 s, 60°C for 30 s, and 72°C for 45 s. The relative quantifications of the mRNAs were normalized to GAPDH using the 2 ^-ΔΔCt^ method. The following gene-specific primer sequences were utilized for the qPCR: IFNB1, 5’-TTGTTGAGAACCTCCTGGCT-3’ and 5’-TGACTATGGTCCAGGCACAG-3’; RANTES, 5’-GGCAGCCCTCGCTGTCATCC-3’ and 5’-GCAGCAGGGTGTGGTGTCCG-3’; ISG15, 5’-CGCAGATCACCCAGAAGATCG-3’ and 5’-TTCGTCGCATTTGTCCACCA-3’ and GAPDH, 5’-GAGTCAACGGATTTGGTCGT-3’ and 5’-GACAAGCTTCCCGTTCTCAG-3’; for Semi-quantitative PCR: IFNB1, 5’-CTCTCCTGTTGTGCTTCTCCA-3’ and 5’-CTCTGACTATGGTCCAGGCAC-3’; RANTES, 5’-ATGAAGGTCTCCGCGGCACGCCT-3’ and 5’-CTAGCTCATCTCCAAAGAGTTG-3’; GAPDH, 5’-GAGAAGGCTGGGGCTCATTT-3’ and 5’-GTCAAAGGTGGAGGAGTGGG -3’.

### Coimmunoprecipitation and Immunoblotting Analyses

The coimmunoprecipitation analyses were performed as previously described [41]. For the transient transfection coimmunoprecipitation experiments, the 293 cells were transfected with the appropriate plasmid. Twenty-four hours after transfection, the cells were harvested and lysed in 1 ml of lysis buffer (20 mM Tris, pH 7.5, 150 mM NaCl, 1% Triton, 1 mM EDTA, 10 μg/ml aprotinin, 10 μg/ml leupeptin, and 1 mM phenylmethylsulfonyl fluoride). For each immunoprecipitation reaction, 0.4 ml of cell lysate was incubated with 0.5 μg of the indicated antibody or control IgG and 40 μl of protein G agarose beads (Santa Cruz Biotechnology, Inc.) at 4°C. After a 4-hour incubation, the beads were washed three times with 1 ml of lysis buffer containing 0.5 M NaCl. The immunoprecipitates and other samples were subjected to SDS-PAGE, transferred onto nitrocellulose membranes and blotted as described previously [67]. For the endogenous coimmunoprecipitation experiments, the 293 cells were treated with or without SeV for the indicated times. The subsequent procedures were performed as described above.

### Native PAGE

To examine the IRF3 dimerization, native PAGE assays were performed as previously described [59]. Briefly, 293 cells were harvested and lysed with ice-cold lysis buffer (50 mM Tris-HCl, pH 7.5, 150 mM NaCl, 1 mM PMSF and 0.5% NP-40). Next, the cell lysates were diluted with 2× loading buffer (125 mM Tris/HCl, pH 6.8, 30% glycerol and 0.1% bromophenol blue). Finally, the samples were loaded onto 15×1.0 mm precast 7.5% native gels and separated at 20 mA for 90 minutes by electrophoresis. The subsequent immunoblotting analyses were performed as described above.

### RNAi Experiments

According to the pSUPER RNAi System manual (OligoEngine, Inc.), double-strand oligonucleotides corresponding to the target gene were cloned into pSUPER vectors. The target sequences for the human DYRK2 gene were the following: #1: 5’-GAGCTCATCAAGAAGAATA-3’; #2: 5’-GGACAGTGCTCACGACACA-3’; and #3: 5’-GGTGCTATCACATCTATAT-3’.

### RNAi-Transduced Stable THP-1 Cells

Control or DYRK2-RNAi retroviral plasmids were co-transfected with packaging plasmids (pGAG-Pol and pVSV-G) into 293 cells. Twenty-four hours after transfection, the cell culture medium was replaced with new medium without antibiotics, and then the cells were incubated for 24 h. The THP-1 cells were then infected with recombinant virus-containing medium in the presence of polybrene (8 g/mL) and were selected with puromycin (0.5 g/mL) for one month before additional experimentation.

### Viral Plaque Assay

RNAi-transduced stable THP-1 cells or 293 cells transfected with the indicated plasmids for 20 h were infected with VSV (MOI = 0.1). One hour after infection, the cells were washed with PBS three times, and medium was then added for another 24 h of incubation. The supernatants were diluted by 10^−6^ to infect Vero cells seeded in 24-well plates. After 1 h infection, 3% methylcellulose was overlaid, and the plates were incubated for 2 days. The overlay was removed, and cells were fixed with 4% paraformaldehyde for 20 min and stained with 1% crystal violet for 30 min. The plaques were counted, averaged, and multiplied by the dilution factor to determine the viral titer as pfu/ml.

### ELISA

RNAi-transduced stable THP-1 cells or 293 cells transfected with the indicated plasmids for 20 h were infected with HSV-1 or SeV. Twelve hours after infection, the culture medium was collected for quantitation of the IFN beta with an ELISA kit (Pestka Biomedical Laboratories) following the protocols recommended by the manufacturer.

### Accession Numbers

The UniProtKB/Swiss-Prot accession numbers (parentheses) are indicated for the following proteins mentioned in the text: DYRK2 (Q92630), TBK1 (Q9UHD2), NLRP4 (Q96MN2), and DTX4 (Q9Y2E6).

## Supporting Information

S1 Fig Effects of DYRK2 overexpression on the virus-induced activations of the IFN-β promoter, ISRE and NF-κB reporter in HeLa cells. (A, B and C) DYRK2 inhibited the SeV-induced activations of the IFN-β promoter (A) and the ISRE (B) but not the NF-κB reporter (C) in dose-dependent manners in HeLa cells. HeLa cells (1×10^5^) were transfected with the IFN-β promoter, ISRE or NF-κB reporter luciferase plasmids (0.1 μg) and the indicated amounts of DYRK2 expression plasmid. Twenty hours after transfection, the cells were infected with or without SeV for 10 h before the luciferase assays were performed. The graphical data are presented as the means ± the SDs (n = 3). (D, E and F) DYRK2 inhibited the HSV-1-induced activations of the IFN-β promoter (D) and the ISRE (E) but not the NF-κB reporter (F) in dose-dependent manners in the HeLa cells. HeLa cells (1×10^5^) were transfected with the IFN-β promoter, ISRE or NF-κB reporter luciferase plasmids (0.1 μg) and the indicated amounts of DYRK2 expression plasmid. Twenty hours after transfection, the cells were infected or not infected with HSV-1 for 10 h before the luciferase assays were performed. The graphical data are presented as the means ± the SDs (n = 3).
(TIF)

S2 Fig Effects of DYRK2 RNAi on the virus-induced activations of the IFN-β promoter, ISRE and NF-κB Reporter in HeLa cells. (A, B and C) Effects of DYRK2 RNAi on the SeV-induced activations of the IFN-β promoter (B), ISRE (C) and NF-κB reporter (D). HeLa cells (1×10^5^) were transfected with the IFN-β promoter, ISRE or NF-κB reporters (0.05 μg) and the indicated RNAi plasmids (0.5 μg each) for 36 h and then infected or not infected with SeV for 10 h before the luciferase assays were performed. The graphical data are presented as the means ± the SDs (n = 3). (D, E and F) Effects of DYRK2 RNAi on the HSV-1-induced activations of the IFN-β promoter (B), ISRE (C) and NF-κB reporter (D). HeLa cells (1×10^5^) were transfected with the IFN-β promoter, ISRE or NF-κB reporters (0.05 μg) and the indicated RNAi plasmids (0.5 μg each) for 36 h and then infected with or without HSV-1 for 10 h before luciferase assays were performed. The graphical data are presented as the means ± the SDs (n = 3).
(TIF)

S3 Fig Effects of DYRK2 on TBK1 and their mutants on IFN-induction. (A) TBK1 S527D and S527E are constitutively ubiquitinated and degraded. The 293 cells (2×10^6^) were transfected with the indicated plasmids and then treated and analyzed as described in Fig 6C. (B) Overexpression of DYRK2 inhibited TBK1-mediated signaling but the signaling mediated by its mutant. The 293 cells (1×10^5^) were transfected with DYRK2 or control plasmids (0.1 μg) together with IFN-β luciferase and the indicated plasmids (0.1 μg each). Luciferase assays were performed 20 h after transfection. The graphical data are presented as the means ± the SDs (n = 3). (C) Effects of DYRK2 on TBK1- and TBK1 mutant-induced transcriptions of IFNB1, RANTES and ISG15 in 293 cells. The 293 cells (2×10^5^) were transfected with the indicated plasmids for 24 h before qPCR analysis. (D) Overexpression of DYRK2 increases VSV replication. The 293 cells were transfected with the indicated plasmids for 20 h before the cells were infected with VSV (MOI = 0.1). The supernatants were harvested 24 h after infection and used for standard plaque assays.
(TIF)
