# Supplementary figures and images for: DYRK2 Negatively Regulates Type I Interferon Induction by Promoting TBK1 Degradation via Ser527 Phosphorylation
Source: PLoS Pathog. 2015 Sep 25;11(9):e1005179. doi: 10.1371/journal.ppat.1005179 (PMC4583546; doi:10.1371/journal.ppat.1005179)

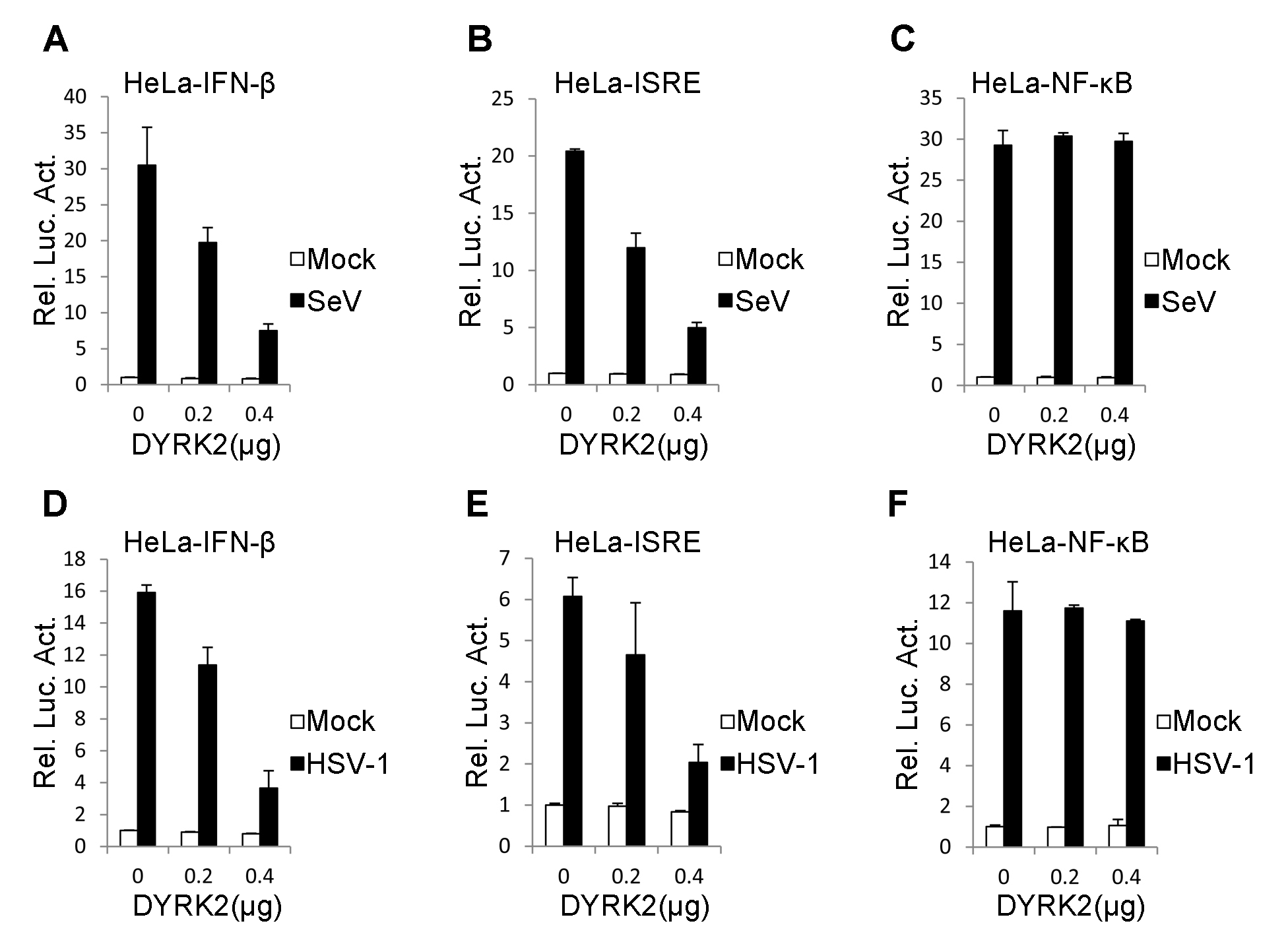

Supplement: S1 Fig — (A, B and C) DYRK2 inhibited the SeV-induced activations of the IFN-β promoter (A) and the ISRE (B) but not the NF-κB reporter (C) in dose-dependent manners in HeLa cells. HeLa cells (1×10^5^) were transfected with the IFN-β promoter, ISRE or NF-κB reporter luciferase plasmids (0.1 μg) and the indicated amounts of DYRK2 expression plasmid. Twenty hours after transfection, the cells were infected with or without SeV for 10 h before the luciferase assays were performed. The graphical data are presented as the means ± the SDs (n = 3). (D, E and F) DYRK2 inhibited the HSV-1-induced activations of the IFN-β promoter (D) and the ISRE (E) but not the NF-κB reporter (F) in dose-dependent manners in the HeLa cells. HeLa cells (1×10^5^) were transfected with the IFN-β promoter, ISRE or NF-κB reporter luciferase plasmids (0.1 μg) and the indicated amounts of DYRK2 expression plasmid. Twenty hours after transfection, the cells were infected or not infected with HSV-1 for 10 h before the luciferase assays were performed. The graphical data are presented as the means ± the SDs (n = 3). (TIF) [file ppat.1005179.s001.tif]

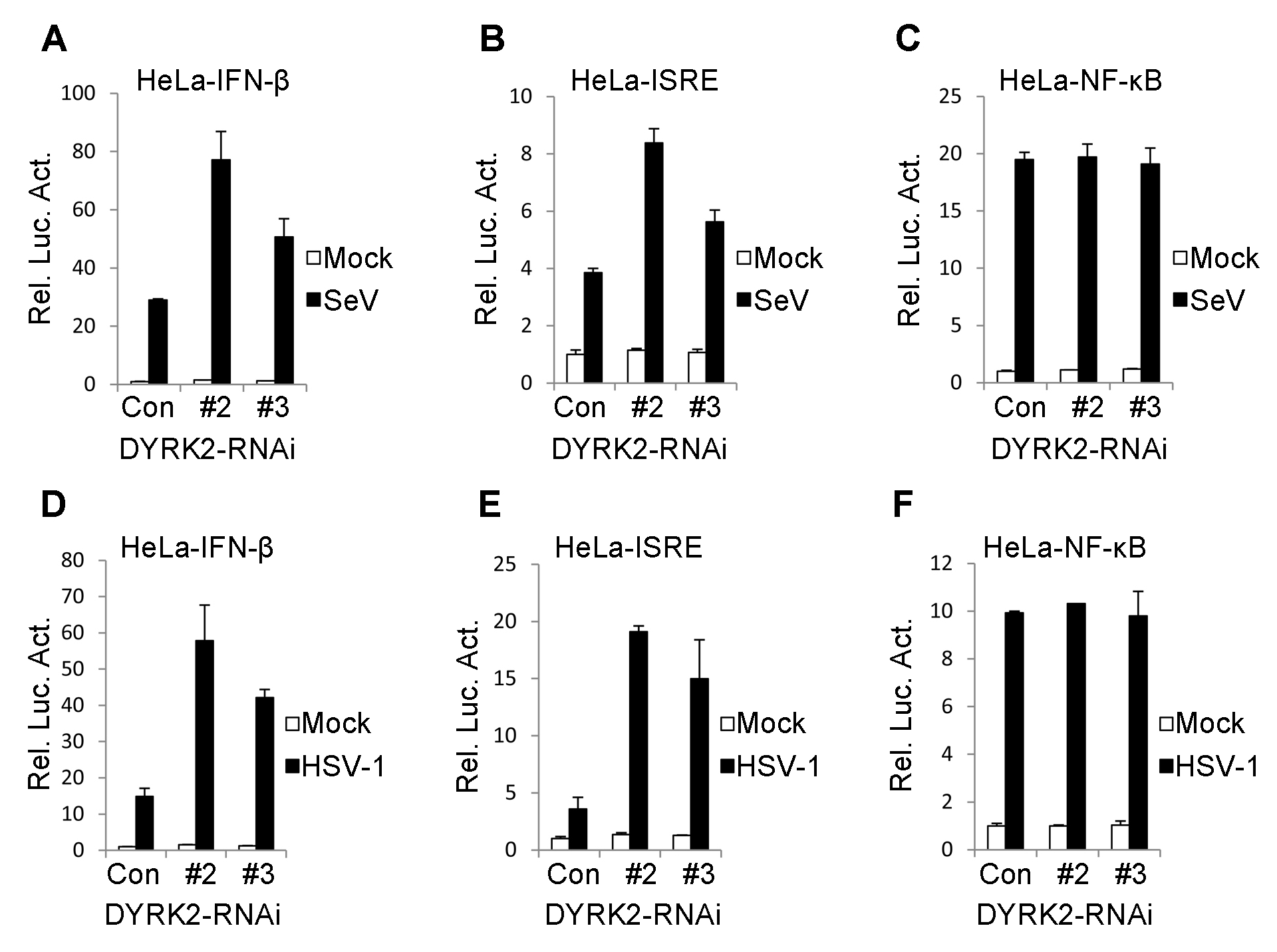

Supplement: S2 Fig — (A, B and C) Effects of DYRK2 RNAi on the SeV-induced activations of the IFN-β promoter (B), ISRE (C) and NF-κB reporter (D). HeLa cells (1×10^5^) were transfected with the IFN-β promoter, ISRE or NF-κB reporters (0.05 μg) and the indicated RNAi plasmids (0.5 μg each) for 36 h and then infected or not infected with SeV for 10 h before the luciferase assays were performed. The graphical data are presented as the means ± the SDs (n = 3). (D, E and F) Effects of DYRK2 RNAi on the HSV-1-induced activations of the IFN-β promoter (B), ISRE (C) and NF-κB reporter (D). HeLa cells (1×10^5^) were transfected with the IFN-β promoter, ISRE or NF-κB reporters (0.05 μg) and the indicated RNAi plasmids (0.5 μg each) for 36 h and then infected with or without HSV-1 for 10 h before luciferase assays were performed. The graphical data are presented as the means ± the SDs (n = 3). (TIF) [file ppat.1005179.s002.tif]

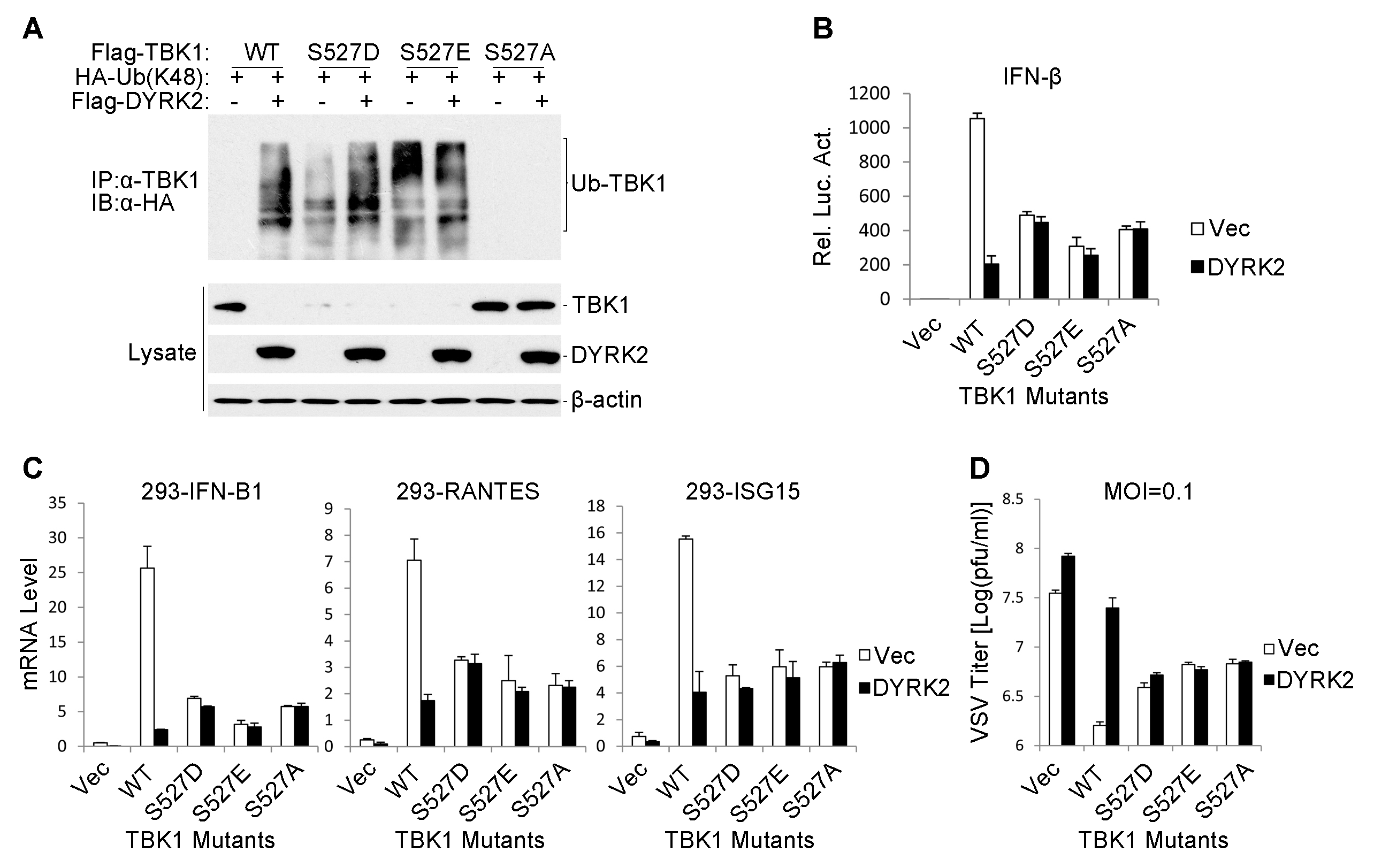

Supplement: S3 Fig — (A) TBK1 S527D and S527E are constitutively ubiquitinated and degraded. The 293 cells (2×10^6^) were transfected with the indicated plasmids and then treated and analyzed as described in Fig 6C. (B) Overexpression of DYRK2 inhibited TBK1-mediated signaling but the signaling mediated by its mutant. The 293 cells (1×10^5^) were transfected with DYRK2 or control plasmids (0.1 μg) together with IFN-β luciferase and the indicated plasmids (0.1 μg each). Luciferase assays were performed 20 h after transfection. The graphical data are presented as the means ± the SDs (n = 3). (C) Effects of DYRK2 on TBK1- and TBK1 mutant-induced transcriptions of IFNB1, RANTES and ISG15 in 293 cells. The 293 cells (2×10^5^) were transfected with the indicated plasmids for 24 h before qPCR analysis. (D) Overexpression of DYRK2 increases VSV replication. The 293 cells were transfected with the indicated plasmids for 20 h before the cells were infected with VSV (MOI = 0.1). The supernatants were harvested 24 h after infection and used for standard plaque assays. (TIF) [file ppat.1005179.s003.tif]
